# Development of models to predict 10-30-year cardiovascular disease risk using the Da Qing IGT and diabetes study

**DOI:** 10.1186/s13098-023-01039-4

**Published:** 2023-03-30

**Authors:** Fei Chen, Jinping Wang, Xiaoping Chen, Liping Yu, Yali An, Qiuhong Gong, Bo Chen, Shuo Xie, Lihong Zhang, Ying Shuai, Fang Zhao, Yanyan Chen, Guangwei Li, Bo Zhang

**Affiliations:** 1 Department of Endocrinology, China-Japan Friendship Hospital, Beijing, China; 2 Department of Cardiology, Da Qing First Hospital, Da Qing, China; 3 Endocrinology Centre, Fuwai Hospital, Chinese Academy of Medical Sciences and Peking Union Medical College, Beijing, China; 4 Division of Non-Communicable Disease Control and Community Health, Chinese Center for Disease Control and Prevention, Beijing, China; 5 Institute of Clinical Medical Sciences, China-Japan Friendship Hospital, Chinese Academy of Medical Sciences & Peking Union Medical College, Beijing, China

**Keywords:** Cardiovascular risk, Risk equation models, Type 2 diabetes

## Abstract

**Background:**

This study aimed to develop cardiovascular disease (CVD) risk equations for Chinese patients with newly diagnosed type 2 diabetes (T2D) to predict 10-, 20-, and 30-year of risk.

**Methods:**

Risk equations for forecasting the occurrence of CVD were developed using data from 601 patients with newly diagnosed T2D from the Da Qing IGT and Diabetes Study with a 30-year follow-up. The data were randomly assigned to a training and test data set. In the training data set, Cox proportional hazard regression was used to develop risk equations to predict CVD. Calibration was assessed by the slope and intercept of the line between predicted and observed probabilities of outcomes by quintile of risk, and discrimination was examined using Harrell’s C statistic in the test data set. Using the Sankey flow diagram to describe the change of CVD risk over time.

**Results:**

Over the 30-year follow-up, corresponding to a 10,395 person-year follow-up time, 355 of 601 (59%) patients developed incident CVD; the incidence of CVD in the participants was 34.2 per 1,000 person-years. Age, sex, smoking status, 2-h plasma glucose level of oral glucose tolerance test, and systolic blood pressure were independent predictors. The C statistics of discrimination for the risk equations were 0.748 (95%CI, 0.710–0.782), 0.696 (95%CI, 0.655–0.704), and 0.687 (95%CI, 0.651–0.694) for 10-, 20-, and 30- year CVDs, respectively. The calibration statistics for the CVD risk equations of slope were 0.88 (*P* = 0.002), 0.89 (*P* = 0.027), and 0.94 (*P* = 0.039) for 10-, 20-, and 30-year CVDs, respectively.

**Conclusions:**

The risk equations forecast the long-term risk of CVD in patients with newly diagnosed T2D using variables readily available in routine clinical practice. By identifying patients at high risk for long-term CVD, clinicians were able to take the required primary prevention measures.

**Supplementary Information:**

The online version contains supplementary material available at 10.1186/s13098-023-01039-4.

## Background

Reducing the cardiovascular disease (CVD) burden in diabetes mellitus is a major clinical imperative that should be prioritized in order to reduce premature death, improve quality of life, reduce individual economic burdens of associated morbidities, and reduce the high cost of medical care [1]. CVD is a major cause of mortality and disability in diabetes, especially in those with type 2 diabetes (T2D) who have a 2–4 fold increase in the risk for CVD when compared with the general population without diabetes [2]. Therefore, risk prediction models are needed for identifying patients with T2D at high risk for CVD, which is an important strategy in the primary prevention of CVD.

Several CVD risk prediction models for patients with T2D have been developed to assist clinicians in estimating patient CVD risk and tailoring management to the needs of patients. Most of these models, however, were developed using data from predominantly Caucasian participants and do not perform well when applied to Chinese patients with T2D due to the ethnic differences in the prevalence of CVD events [3, 4]. For example, the Chinese population has a lower risk of coronary heart disease and heart failure than Caucasians, but a higher risk of stroke [5]. Such differences in prevalence may be explained by the differences in lifestyle behaviours, genetic factors, and environmental influences [6]. Moreover, a study highlighted that the risk prediction models developed from studies in one country or ethnic population might not be suitable for another country or ethnic population; therefore, localised risk prediction models should be developed [7]. Although some CVD risk prediction models have been published for Chinese with T2D, these models are inadequate because they focused on 5-year or 10-year follow-ups with a modest discriminative ability [8, 9]. Therefore, a robust model to accurately predict long-term CVD risk in Chinese T2D is still lacking and urgently needed to enable accurate risk stratification and management to prevent CVD complications in the world’s largest T2D population. Therefore, this study aimed to develop models to predict the 10-, 20-, and 30-year risk of CVD using datasets from the Da Qing IGT and Diabetes Study.

## Methods

### Study design and participants

The design and methods used in the Da Qing IGT and Diabetes Study have been reported elsewhere [10–12]. Briefly, 110,660 residents aged 25–74 years were selected as eligible for resident screening for diabetes in 1986. Finally, 3,956 participants received a 75-g oral glucose tolerance test (OGTT) which included the measurements of plasma glucose concentrations at fasting, after 1-h, and after 2-h. Based on the WHO criteria of 1985 [13], 630 participants were identified as newly diagnosed type 2 diabetes (T2D). The participants were required to undergo a baseline examination that included systolic (SBP) and diastolic (DBP) blood pressure, body mass index (BMI), a 12-lead electrocardiogram, plasma lipids, and OGTT. Details of the baseline examination were previously described [14, 15]. All the newly diagnosed T2D were informed of their diagnosis and received a guideline of available clinical treatment in the local clinic. Written informed consent was obtained from all study participants and proxy informants for the deceased. The study was approved by the WHO and China-Japan Friendship Hospital’s Institutional Review Board.

Of the 630 newly diagnosed T2D participants who underwent baseline examination in 1986, we excluded 19 who had missing information at baseline examination, and 10 with a known history of cardiovascular disease at enrolment. Finally, 601 participants were included in the study.

### Follow-up and cardiovascular events

All participants were tracked from their enrolment to the onset of CVD. Data were collected by personal interview, clinical examination, by trained staff for living participants, while a living spouse, sibling, or child were interviewed for the deceased with standardised questionnaires for the proxy informants. Those unable to attend the hospital because of ill health or living outside of Da Qing city were examined at home, interviewed by telephone, and examined in local hospitals. Data were then verified by review of the medical records and death certificate. CVD events were defined as the first occurrence of non-fatal or fatal myocardial infarctions, sudden death, and non-fatal or fatal stroke. The earliest date of recognition of the CVD event from medical records, interviews, or the 20- and 30-year follow-up examinations was used to define the onset of CVD. First occurrence of CVD of 355 participants were reported by December 31, 2016. Moreover, we could infer the CVD status at the 10-year follow-up based on the onset date of CVD.

### Statistical analysis

Participants’ characteristics are shown as the mean (± standard deviation) for quantitative parameters and as a percentage for categorical variables. Descriptive statistics were compared between participants who developed CVD or never developed CVD within a 30-year follow-up. ANOVA tests and ${\chi }^{2}$ tests were used for normally distributed continuous variables and categorical variables, separately. CVD incidence rates were calculated by dividing the sum of the events by the sum of person-years. The participants’ follow-up person-years were calculated from date of enrolment to the first onset of CVD.

The data were randomly assigned to two subsamples of roughly equal sizes: the training dataset (n = 300) and the test dataset (n = 301). Cox proportional hazard regression with the step forward algorithm was used to select predictors at baseline for incident CVD as long as the Akaike information criterion fell by at least the number of extra parameters. Finally, the prediction model included the baseline variables of age, sex, smoking status, plasma glucose levels 2 h after the oral glucose tolerance test (2 h-PG), and SBP. All the continuous variables were naturally logarithmically transformed to improve the discrimination and calibration of the models and to minimise the influence of extreme observations.

Based on Cox proportional hazard regression, the risk score was =$${X}_{1}\times {\beta }_{1}+{X}_{2}\times {\beta }_{2}\cdots +{X}_{n}\times {\beta }_{n}$$. The probability of CVD over j years was: CVD risk probability = $$1-{S\left(j\right)}^{\text{exp}\left(risk score-mean of the risk score\right)},$$ where $${X}_{1},{ X}_{2},{\cdots X}_{n}$$ were baseline predictors and $${\beta }_{1},{\beta }_{2},{\cdots \beta }_{n}$$ were the estimated coefficients of baseline predictors, and $$S\left(j\right)$$ was the survival probability over j years when the risk equation took the value of its mean.

We evaluated the ability of the risk prediction model to discriminate participants who experience a CVD event from those who do not, using an overall C statistic [16] in the test set, expanding on a suggestion by Harrell et al. [17]. The C statistic is analogous to the area under the receiver-operating characteristic curve. Bootstrapping was performed 200 times for the estimation of the 95% confidence intervals for the C statistic. We evaluated the calibration through the slope and intercept of the line between predicted and observed probabilities of each outcome by the quintile of risk [18].

Sankey flow diagrams [19] is a data visualisation technique that emphasises flow/movement/change from one state to another or one time to another, which is popular in economics, business, and science to examine complex multi-step processes. We used the Sankey flow diagrams to visualise the CVD risk of patients over time to identify patients at high risk who needed primary prevention.

R software version 4.1.0 were used for all statistical analyses. A 2-tailed with *P* < 0.05 was set for the statistical significance level.

## Results

### Baseline characteristics

During the 30-year follow-up, corresponding to 10,395 person-years of follow-up time, 59% (355/601) of participants had a first CVD incident, conferring an incidence of 34.2 per 1,000 person-years. The mean age was 48.3 years (SD = 8.7), and 52.1% (313/601) participants were females. First CVDs were occurred in 107 (18%), 271 (45%), and 355 (59%) participants of 10-, 20-, and 30-year follow-ups, respectively. The baseline characteristics of people who progressed to CVD or never developed CVD within the 30-year follow-up were shown in Table 1. The participants who developed CVD were more likely to be male, older, with elevated CVD risk profiles such as smoking, elevated plasma glucose levels 1 h after the oral glucose tolerance test, and elevated SBP and DBP.

**Table 1:** Baseline characteristics of participants who progressed to CVD or never developed CVD within the 30-year follow-up

	No CVD	Incident CVD	*P*
N (%)	246 (40.9%)	355 (59.1%)	
Sex (male, %)	104 (42.3%)	184 (51.8%)	0.026
Age (years)	46.9 (9.78)	49.2 (7.79)	0.001
BMI (kg/${m}^{2}$)	25.2 (3.70)	25.8 (3.55)	0.051
Current smoker (%)	71 (28.9%)	138 (38.9%)	0.014
Fasting plasma glucose (mmol/L)	8.3 (2.9)	8.8 (3.1)	0.055
1 h-PG (mmol/L)	15.7 (3.4)	16.3 (3.5)	0.029
2 h-PG (mmol/L)	15.0 (3.5)	15.5 (3.7)	0.153
Systolic Blood Pressure (mm Hg)	131.9 (22.0)	138.0 (24.8)	0.002
Diastolic Blood Pressure (mm Hg)	85.7 (13.8)	89.8 (14.6)	0.001

Data presented as mean (SD) for continuous variables or n (%) for categorical variables. CVD, cardiovascular disease; BMI, body mass index; 1 h-PG /2 h-PG, venous plasma glucose concentration 1 and 2 h after 75 g oral glucose load, respectively

### Developing CVD risk equations in participants with newly diagnosed T2D

The multivariable-adjusted regression coefficients and hazard ratios for incident CVD events were presented in Table 2. The CVD risk equations included standard cardiovascular risk factors such as age, sex, smoking status, 2 h-PG, and SBP. We observed statistically significant relations of most risk factors in Table 2. The developed CVD risk score was 0.26 × sex (1 for male) + 2.36 × log_e_ (age) + 0.22 × current smoker (1 for yes) + 0.94 × log_e_ (OGTT 2h plasma glucose ) + 1.37 × log_e_ (systolic blood pressure). The average survival ${s}_{0}$ at 10-, 20-, and 30- year follow-up times were 0.812, 0.487, and 0.279, respectively. The risk probability of CVD = $1-{s}_{0}^{exp(risk score-8.21)}$. The discrimination and calibration of CVD risk equations were moderate. The C statistics of discrimination for the risk equations were 0.748 (95%CI, 0.710–0.782), 0.696 (95%CI, 0.655–0.704), and 0.687 (95%CI, 0.651–0.694) for 10-, 20-, and 30-year CVDs, respectively. The calibration statistics for the CVD prediction equations of slope were 0.88 (*P* = 0.002), 0.89 (*P* = 0.027), and 0.94 (*P* = 0.039) for 10-, 20-, and 30-year CVDs, respectively, indicating relative goodness of fit.

**Table 2:** Regression coefficients and hazard ratios for the CVD risk prediction model

Variables	Beta	*P*	Hazard Ratio	95% CI
S0(10) = 0.812				
S0(20) = 0.487				
S0(30) = 0.279				
Sex (male)	0.26	0.028	1.3	(1.02–1.64)
Log of age	2.36	< 0.001	10.58	(5.31–21.07)
Current smoker	0.22	0.076	1.24	(0.98–1.58)
Log of 2 h-PG	0.94	< 0.001	2.56	(1.63–4.02)
Log of systolic blood pressure	1.37	< 0.001	3.95	(2.01–7.78)

S0(10), the average survival probability of the participants in 10-year; S0(20), the average survival probability of the participants in 20-year; S0(30), the average survival probability of the participants in 30-year

### The progression of CVD risk over time

**Fig. 1:**
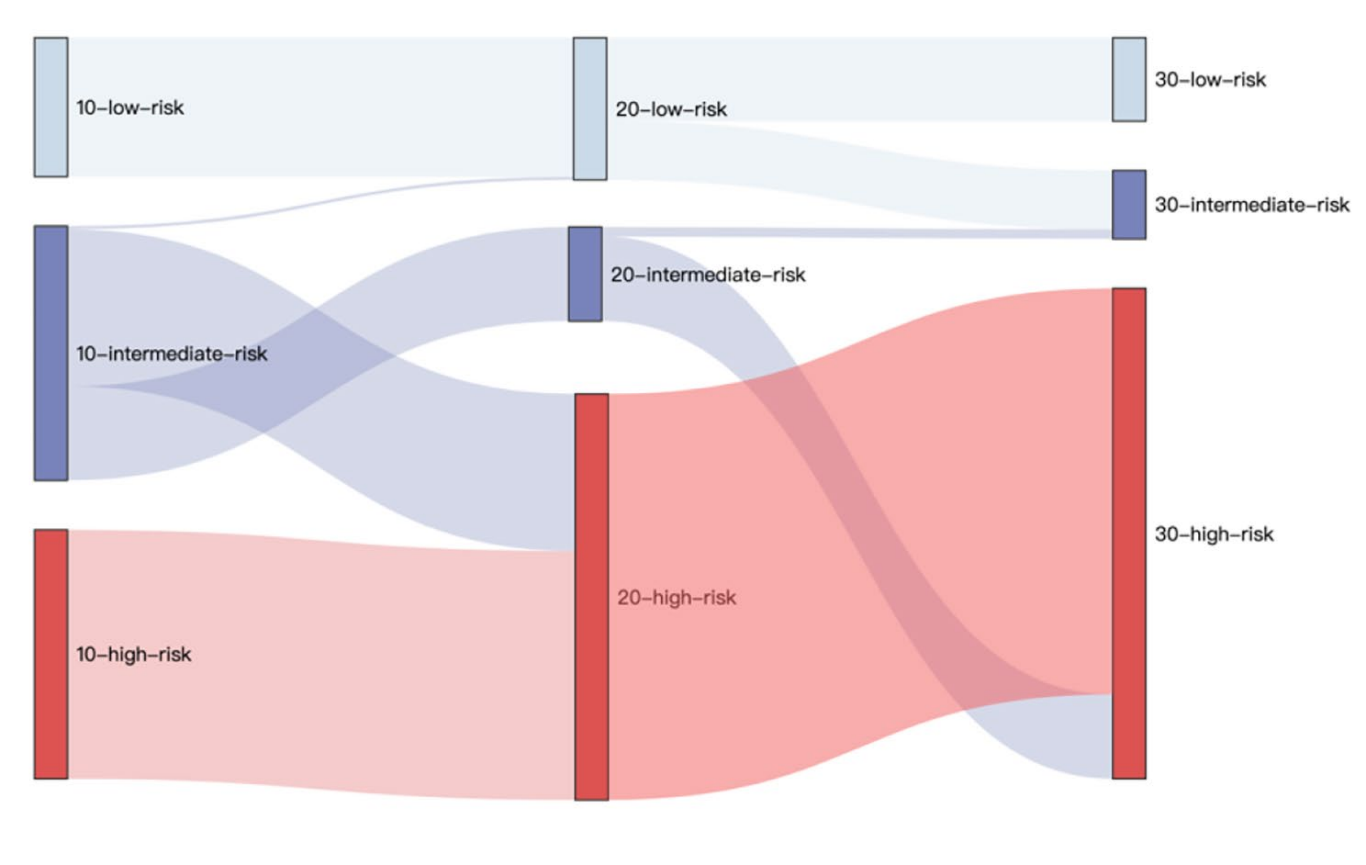
Changes in the participants with low, intermediate, and high risks of CVD over time. 10-low-risk, 130 participants with the lower risk of CVD events in a 10-year follow-up in which the risk score is < 10%; 10-intermediate-risk, 238 participants with the intermediate risk of CVD events in a 10-year follow-up in which the risk score is between 10% and 20%; 10-high-risk, 233 participants with the higher risk of CVD events in a 10-year follow-up in which the risk score is > 20%. The definitions of 20-low-risk, 20-intermediate-risk, 20-high-risk are similar with the 10-year with the thresholds of < 35%, 35-45%, > 45%, separately. There are 380 participants in 20-high-risk groups, 88 participants in 20-intermediate-risk group, 133 participants in 20-low-ris group. In the same way, 459 participants with the risk score > 60% were assigned to the 30-high-risk group, 64 participants with the risk score between 50% and 60% were assigned to the 30-intermediate-risk group, and 78 participants with the risk score < 50% were assigned to the 30-low-risk group

Through the CVD risk equations developed in this study, the CVD risk score could be calculated for every participant in 10-, 20-, and 30-year follow-ups, respectively. Based on the risk population proportion [20] and the prevalence of CVD with 18% (107/601) in 10-year follow-ups in this study, participants with a 10-year CVD risk score ≥ 20% were assigned to the high-risk group; those with a 10-year CVD risk score of < 10% were assigned to the low-risk group based on the published study [21]; and those with a risk score between 10% and 20% were assigned to the intermediate-risk group (Fig. 1).

We repeated a similar method while processing CVD risk scores of 20- and 30-year follow-ups. The prevalence of CVD with 45% in 20-year follow-ups in this study, therefore, participants with a 20-year CVD risk score ≥ 45% were assigned to high-risk group. The CVD risk score of 20 years with thresholds < 35%, and 35–45% were assigned to the low-risk, intermediate-risk based on the risk population proportion [20], respectively. In the same way, the CVD risk score of 30 years with the thresholds < 50%, 50–60%, and > 60% were assigned to the low-risk, intermediate-risk, and high-risk groups, respectively (Fig. 1).

Based on the definitions of high-, intermediate-, and low- risk of 10-year CVD risk score, 233 participants, 238 participants, and 130 participants were assigned to 10-high-risk group, 10-intermediate-risk group, and 10-low-risk group, separately. Meanwhile, there were 380 participants in the 20-high-risk group, 88 participants in the 20-intermediate-risk group, and 133 participants in the 20-low-risk group. Moreover, 459 participants were assigned to 30-high-risk group, 64 participants were assigned to 30-intermediate-risk group, and 78 participants were assigned to 30-low-risk group. Through the number of high-risk participants, the CVD risk equations may overestimate the risk of CVD in newly diagnosed T2D population. Through the Sankey flow diagrams in Fig. 1, the high-risk group (n = 233) in 10-year follow-up remained high risk over 20- and 30-year follow-ups and needed to take primary prevention when diagnosed, followed by 235 of 238 participants in the intermediate-risk group who developed high risk in 30-year follow-ups and were overlooked in published CVD risk models because of not high-risk participants in risk models.

## Discussion

We developed risk equations to predict 10-, 20-, and 30-year CVD risk in participants with newly diagnosed T2D aged 25–74 years using data commonly available in clinical practice, such as age, sex, smoking status, 2 h-PG, and SBP. To our knowledge, these are the first long-term CVD risk equations developed in China. They are based on longer follow-up data and more comprehensively capture the progression of CVD. Our risk equations are based on newly diagnosed T2D patients without previous CVD events, and the models predict the risk of CVD as a primary event.

In terms of model discrimination and calibration, the C statistics were 0.748 (95%CI, 0.710–0.782), 0.696 (95%CI, 0.655–0.704), and 0.687 (95%CI, 0.651–0.694) and the calibration statistics for CVD prediction equations of slope were 0.88 (*P* = 0.002), 0.89 (*P* = 0.027), and 0.94 (*P* = 0.039) for 10-year CVD, 20-year CVD, and 30-year CVD, respectively. Through the Sankey flow diagram, we observed the risk scores of CVD over time and identified patients at high risk of CVD in 30 years for early prevention.

Although the Chinese Multi-provincial Cohort Study and the China-PAR project had developed 10-year CVD risk prediction models for Chinese individuals to guide the prevention of CVD [22, 23], the study populations were the general population and not for individuals with T2D. Therefore, our focus was on T2D to develop the CVD risk prediction equations. Some published CVD-related models focused on the 5-year or 8-year follow-up in Chinese with T2D [3, 24, 25] and cannot predict the long-term CVD risk. However, we developed 10-, 20-, and 30-year CVD risk prediction equations that can predict the long-term CVD risk of patients with T2D. Through the long-term CVD risk prediction equations, we can implement the stratified management and advanced prevention of CVD in patients with newly diagnosed T2D. Treatment target recommendations regarding the risk factor control may need to be more aggressive in participants who have been identified as high-risk for CVD in either 10-, 20-, or 30-year follow-ups. This group of patients was focused on by clinicians and entailed much spending of medical resources. Moreover, patients who have been assigned to intermediate-risk for 10-year CVD risk and developed high risk in 20-, and 30-year periods were advised to maintain a healthy glycaemic level, lose weight, and increase physical activity to lower the risk of CVD with an appropriate expenditure of medical resources. Moreover, participants who were assigned to intermediate risk at 10 and 20 years and developed high-risk at 30 years also needed to step up exercise and to keep glycaemia, blood pressure and lipids on target, and reduce body weight if obese. To some extent, with the help of the CVD risk equations, patients with newly diagnosed and long-standing diabetes can reduce their CVD risk by maximizing the utilization of clinical resources [26, 27]. From a national perspective, China has the highest number of people with diabetes worldwide, and patients with diabetes who were assigned to low- and intermediate-risk at 30 years were advised to maintain their existing drug treatments and lifestyle, and other patients took precision treatments. In this way, the CVD risk equation can reduce national health insurance costs.

We observed differences of 1 h-PG in baseline between participants who developed CVD and did not develop CVD. Moreover, the performance of the predictive models using 2 h-PG or 1 h-PG as a predictor was similar (Additional file 1: Table S1 ) which demonstrated that 1 h-PG also needed strict control. In addition, some studies showed that 1 h-PG predictive performance was similar to 2 h-PG in the prediction of T2D, complications and mortality [28, 29]. Based on the evidence from this study and the results of published studies [28, 29], we showed the 1 h-PG as a predictor in additional file 1 Table S1 . This study emphasized the significance of 1 h-PG, which was frequently ignored and undervalued by clinicians. To facilitate the promotion of the model, we also showed the CVD risk equations which included 1 h-PG in additional file 1 Table S2 . It has been established that lipid information, especially low-density lipoproteins, are important predictors for CVD [30, 31]. However, this study lacked the measurement of related indicators which was a major limitation. Without using blood lipid information, our CVD risk prediction models achieved moderate discrimination, which indicated a potentially wider use based on the five easy-access predictors.

Through the performance of CVD risk models, the performance of CVD risk equations of 20-, and 30-years has declined. The possible reason being that the predictive ability of the baseline for long-term CVD was weak, and intermediate variables or drugs need to be added to improve the predictive ability. Although the performance to predict CVD risk at 20- and 30-years was moderate, the study provided a tool for long-term CVD prediction and showed the CVD risk change over time.

This study has some limitations. First, we did not validate the CVD risk prediction models in an external dataset of T2D to evaluate the performance. The external dataset of T2D with a 30-year follow-up was lacking, which limited the validation of CVD risk prediction. Second, the CVD risk equation did not include lipid information because of lacking the measures of low-density lipoproteins and the 25% missing rate of triglyceride; however, the performance of the risk equation was good and may have a wider application in clinical management. Third, although the smoking status was not significant in the model, the smoking status was included in the CVD risk equation model which was very important for CVD events. Smoking status was a risk factor for CVD events in the univariate model, however, after adjusting for age and sex, smoking status was not significant risk factor for CVD events. The reason for the smoking status not being significant was that sex, age and smoking status have some degree of correlation. Future research will verify the performance of this model in an external validation set, and further promote this model for clinical use.

## Conclusions

This study developed long-term CVD risk equations for Chinese patients with newly diagnosed T2D with a 30-year follow-up. It offers a useful tool for the clinician faced with the increasing prevalence of CVD in T2D. It will aid decision-making to provide early appropriate action to decrease the risk of adverse outcomes as well as aid health service planning.

## Electronic Supplementary Material

Below is the link to the electronic supplementary material.

Supplementary Material 1

## Data Availability

The dataset used in the study is available from the corresponding author upon reasonable request.
