# Correction to: Development of models to predict 10-30-year cardiovascular disease risk using the Da Qing IGT and diabetes study

**DOI:** 10.1186/s13098-023-01066-1

**Published:** 2023-05-02

**Authors:** Fei Chen, Jinping Wang, Xiaoping Chen, Liping Yu, Yali An, Qiuhong Gong, Bo Chen, Shuo Xie, Lihong Zhang, Ying Shuai, Fang Zhao, Yanyan Chen, Guangwei Li, Bo Zhang

**Affiliations:** 1 Department of Endocrinology, China-Japan Friendship Hospital, Beijing, China; 2 Department of Cardiology, Da Qing First Hospital, Da Qing, China; 3 Endocrinology Centre, Fuwai Hospital, Chinese Academy of Medical Sciences and Peking Union Medical College, Beijing, China; 4 Division of Non-Communicable Disease Control and Community Health, Chinese Center for Disease Control and Prevention, Beijing, China; 5 Institute of Clinical Medical Sciences, China-Japan Friendship Hospital, Chinese Academy of Medical Sciences & Peking Union Medical College, Beijing, China

**Correction: Chen et al. Diabetology & Metabolic Syndrome _#####################_**

10.1186/s13098-023-01039-4

Following publication of the original article [1], the authors identified an error in first affiliation of the author. The correct affiliation is given below.

“Department of Endocrinology, China-Japan Friendship Hospital, Beijing, China.”.

The original article has been corrected.

