# Supplementary material for: Development of models to predict 10-30-year cardiovascular disease risk using the Da Qing IGT and diabetes study
Source: Diabetol Metab Syndr. 2023 Mar 30;15:62. doi: 10.1186/s13098-023-01039-4 (PMC10061839; doi:10.1186/s13098-023-01039-4)
Supplement: Supplementary file 1 — Supplementary Material 1 [file 13098_2023_1039_MOESM1_ESM.docx]

**Supplementary Information**

Additional file 1

Table S1. The comparison of the performance of the CVD risk equation using 1h-PG and 2h-PG

|  | Discrimination | | |
| --- | --- | --- | --- |
| Model | C statistics of 10 year (95%CI) | C statistics of 20 year (95%CI) | C statistics of 30 year (95%CI) |
| Predictor 1 | 0.734 (0.698–0.764) | 0.678 (0.654–0.701) | 0.672 (0.651–0.692) |
| Predictor 2 | 0.748 (0.710–0.782) | 0.696 (0.655–0.704) | 0.687 (0.651–0.694) |

1h-PG /2h-PG, venous plasma glucose concentration 1 h and 2 h after 75g oral glucose load, respectively; Predictor 1 contains age, sex, smoking status, 1h-PG, and systolic blood pressure; Predictor 2 contains age, sex, smoking status, 2h-PG, and systolic blood pressure.

**Table S2** Regression coefficients and hazard ratios for the CVD risk prediction model using 1h-PG

| variables | beta | *P* | Hazard Ratio | 95% CI |
| --- | --- | --- | --- | --- |
| S0(10) = 0.812 |  |  |  |  |
| S0(20) = 0.487 |  |  |  |  |
| S0(30) = 0.279 |  |  |  |  |
| Sex (male) | 0.38 | 0.022 | 1.46 | (1.06–2.02) |
| Log of age | 2.00 | < 0.001 | 7.38 | (2.96–18.39) |
| Current smoker | 0.17 | 0.319 | 1.18 | (0.85–1.65) |
| Log of 1h-PG | 1.17 | 0.001 | 3.23 | (1.60–6.51) |
| Log of Systolic blood pressure | 1.53 | 0.001 | 2.83 | (1.83–11.65) |

S0(10), the average survival probability of the participants in 10-year; S0(20), the average survival probability of the participants in 20-year; S0(30), the average survival probability of the participants in 30-year.
